# Astaxanthin mitigates cobalt cytotoxicity in the MG-63 cells by modulating the oxidative stress

**DOI:** 10.1186/s40360-017-0166-1

**Published:** 2017-07-24

**Authors:** Dahe Li, Wenwen Tong, Denghui Liu, Yuming Zou, Chen Zhang, Weidong Xu

**Affiliations:** 1 Department of Orthopedics, The Eighty-eighth Military Hospital, Tai’an, 271000 China; 2 Department of Orthopedics, Shanghai Changhai Hospital, Naval Medical University, Shanghai, 200433 China

**Keywords:** Astaxanthin, MG-63 cells, Cobalt cytotoxicity, Oxidative stress

## Abstract

**Background:**

With the re-popularity of metal-on-metal (MoM) bearing in recent years, the cobalt toxicity has been a cause for concern in the total hip replacement surgery by both physicians and patients.

**Methods:**

MG-63 cell line was cultured in vitro and incubated with cobalt (II) chloride (CoCl_2_) and/or with astaxanthin (ASX) for 24 h. MTT assay was conducted to evaluate the cell viability after cobalt exposure and ASX treatment. Fluorescence-activated cell sorting (FACS) analysis was performed to examine the reactive oxygen species (ROS) level. Quantitative real-time polymerase chain reaction (PCR) was adopted to determine the mRNA levels of related targets. And western blot analysis was used to examine the protein expressions. One-way ANOVA with posttest Newman-Keuls multiple comparisons was adopted to analysis all the obtained data.

**Results:**

In the current study, ASX exhibited significant protective effect against the Co(II)-induced cytotoxicity in MG-63 cell line. We also found that ASX protected the cells against Co-induced apoptosis by regulating the expression of Bcl-2 family proteins. Besides, heme oxygenase 1 (HO-1) could be activated by Co exposure; ASX treatment significantly inhibited HO-1 activation, suppressing the oxidative stress induced by Co exposure. Moreover, c-Jun N-terminal Kinase (JNK) phosphorylation was shown to participate in the signaling pathway of the protective effect of ASX. However, knockdown of JNK expression by siRNA transfection or JNK inhibitor SP600125 treatment did not affect the protective effect of ASX against cobalt cytotoxicity in MG-63 cells.

**Conclusions:**

ASX mitigated cobalt cytotoxicity in the MG-63 cells by modulating the oxidative stress. And ASX could be a promising therapy against cobalt toxicity in the hip articulation surgery.

**Electronic supplementary material:**

The online version of this article (doi:10.1186/s40360-017-0166-1) contains supplementary material, which is available to authorized users.

## Background

Hip arthroplasty is the most commonly used therapy to treat joint failure caused by osteoarthritis [1]. Metal-on-metal (MoM) bearings were originally reintroduced over the last 20 years [2] because of their lower volumetric wear rates in comparison to conventional metal-on-polyethylene bearings [3, 4], especially in young and active patients [5]. However, with a MoM hip prosthesis, continuous motion could cause the generation of metal particles and ions [6–9], mainly cobalt (Co) and chromium (Cr), which lead to systemic or local toxicity such as neuro-ocular toxicity, cardiotoxicity, bone loss, tissue damage, metal hypersensitivity and chromosomal changes [10, 11]. Significant increase of Co ion was found in the whole blood or serum of MoM-implanted patients, compared with preoperative values; and the elevated Co level was connected to the head size of the implanted MoM bearings [8, 12, 13]. It’s well known that Co of high concentration is toxic [14]; nevertheless, the potential underlying mechanism might be involved with enhanced tissue oxidative stress [15], triggered intrinsic apoptosis [16, 17], which impact osteoclast activity though inducing CSF (colony stimulating factor) and RANKL (Receptor Activator for Nuclear Factor-κB Ligand), as well as cytokines secretion of microphage cells (TNF-α, IL-6) [18–20].

Astaxanthin (ASX) is a dark red pigment and a dietary carotenoid found in algae, crustaceans, and fish [21, 22]. Despite that it is a primary component of coloration, ASX is a protective agent in marine plants and fish. For example, in fish, dietary ASX could alter liver function and improve defenses against oxidative stress. It has been reported that ASX is 5 to 15 times more potent antioxidant than β-carotene and lutein, which share similar structure with ASX [23]. The mechanism underlying the anti-oxidative effect of ASX included blocking ROS generation and dose-dependently inhibiting apoptosis through a mitochondrial signaling pathway [24]. Besides, some studies also reported that ASX has other potent biological activity such as anti-inflammation, anticancer and immuno-modulation [25]. Barim-Oz et al. reported that ASX is a more potent antioxidant than vitamin C, vitamin A and beta-carotene [26]. Fang et al. also demonstrated that ASX protects against early burn-wound progression in rats by attenuating oxidative stress-induced inflammation and mitochondria-related apoptosis [27]. Kim et al. proved that ASX inhibits inflammation and fibrosis in the liver and adipose tissue of mouse models of diet-induced obesity and nonalcoholic steatohepatitis [28]. Lin et al. found that ASX stimulates immune responses by enhancing IFN-gamma and IL-2 Secretion in primary cultured lymphocytes in vitro and ex vivo [29].

Previous results have demonstrated that Co (II) ions could induce oxidative stress in MG-63 cells, an osteosarcoma cell line [30]. To explore the potential protective effect of antioxidant ASX against oxidative stress in MG-63 cells, the current study examined cell viability, ROS level, apoptosis and secretion of cytokines after Co(II) and ASX treatment.

## Methods

### Materials

Cobalt (II) chloride (CoCl_2_) was purchased from Sigma-Aldrich (St. Louis, MO; #60818). Astaxanthin was purchased from Sigma-Aldrich (St. Louis, MO; #41659). Dimethyl sulfoxide (DMSO) was obtained from Sangon Biotech (Shanghai, China; # A100231).

### Cell culture and treatment

MG-63 was purchased from ATCC (American Type Culture Collection), the cells were cultured in ATCC-formulated Eagle’s Minimum Essential Medium (Hyclone, USA) supplemented with 10% fetal bovine serum (Hyclone, USA) at 37 °C in 5% CO_2_ and subcultured every 2 to 3 days. In 80% conjugation, the cells were starved free of FBS overnight and treated with cobalt (II) of different concentrations (10, 50, 100, 200, 400 μM) for 24 h. For astaxanthin treatment, the concentrations include 1, 5, 10, 20 nM, and the time period was also 24 h.

### Cell viability assay

In vitro MTT (Thiazolyl blue tetrazolium bromide) cell proliferation and cytotoxicity assay was performed. Cells were cultured at 5000 per well in 96-well tissue culture plates. To assess cell viability, cobalt or AST were added after plating. At the end of the culture period, 20 μL of MTT solution (CellTiter 96 Aqueous One Solution Cell Proliferation Assay; Beyotime Biotechnology, China) was added, the cells were incubated for a further 2 h, and the absorbance was measured at 490 nm using an ELISA plate reader (Beyotime Biotechnology, China). Because the absorbance at 490 nm is linear with the cell concentration to a certain degree in the MTT test. The cell viability was calculated using OD490 (optical density) through the following equation:

$$ \mathrm{the}\ \mathrm{cell}\ \mathrm{viability}\ \mathrm{of}\ \mathrm{X}\ \left(\%\right)=\frac{\mathrm{ODx}}{\mathrm{Average}\ \mathrm{OD}\left(\mathrm{control}\ \mathrm{group}\right)}\times 100. $$

### Measurement of intracellular ROS

Fluorescent probe DCFH-DA was used to determine the changes of the intracellular generation of ROS. After treatments, cells were rinsed three times with PBS, and incubated with 5 μM DCFH-DA at 37 °C for 30 min. The fluorescence intensity was examined at 525 nm using Microplate Reader (Thermo Scientific, USA).

### Quantitative real-time polymerase chain reaction (PCR).

The total RNA from the cells was isolated using the TRIzol reagent (Invitrogen), and the cDNA was synthesized following the manufacturer’s protocols using 1 μg RNA (Prime ScriptTM RT reagent Kit, Takara). qRT-PCR was performed using a standard SYBR-green PCR kit (Takara), and the gene-specific PCR amplification was performed using the Applied Biosystems 7300 Sequence Detection system (Applied Biosystems, USA). The qRT-PCR reactions, including the no-template controls, were performed in triplicate. For each sample, the data were normalized to the housekeeping gene glyceraldehyde 3-phosphate dehydrogenase (GAPDH). The designed primers in this study were:

TNF-α forward primer, 5′- GAGGCCAAGCCCTGGT ATG-3′,

 and TNF-α reverse primer, 5′-GAGGCCAAGCCCTGGTATG -3′;

GAPDH forward primer, 5′- ACAACTTTGGTATCGTGGAAGG-3′;

and GAPDH reverse primer, 5′- GCCATCACGCCACAGTTTC-3′;

NF-κB forward primer, 5′- GCTTAGGAGGGAGAGCCCAC -3′;

and NF-κB reverse primer, 5′- TAGGACGTTGTGTTCCTTCCG -3′;

HO-1 forward primer, 5′- ACAACTTTGGTATCGTGGAAGG-3′;

and HO-1 reverse primer, 5′- GCCATCACGCCACAGTTTC-3′.

Analysis was performed using the comparative Ct value method. For each sample, the data were normalized to the housekeeping gene GAPDH.

### Western blot analysis

The cells were lysed in RIPA lysis buffer (Beyotime). The lysates were centrifuged at 12,000 rpm and 4 °C for 10 min. The same amounts of protein were separated using 10–15% odium dodecyl sulfate-polyacrylamide gel electrophoresis (the exact concentration was determined by the molecular weight of the detected proteins) and transferred to nitrocellulose membranes (Millipore). For immunodetection, the membranes were incubated with specific antibodies and the following antibodies were used: anti-Bcl2 (Abcam, ab7973), anti-BAX (Abcam, ab7977), anti-caspase 3(CST, 9662S), anti-HO-1(CST, 5853S), anti-ERK (Abcam, ab17942), anti-p-ERK(CST, 5683S), anti-JNK(CST, 9258S), anti-p-JNK(CST, 9255S), anti-AKT(CST, 4691S), anti-p-AKT(CST, 4060S), anti-P38(CST, 9212S), anti-p-P38(CST, 4511S). The immunoblots were developed using horseradish peroxidase (HRP)-coupled anti-rabbit secondary antibodies (ProteinTech Group) and then detected with enhanced chemiluminescence (Pierce Biotechnology). The GAPDH protein was used as a control.

### Cell apoptosis analysis by FACS

The cells were treated with Co^2+^ or (and) ASX, after incubation for 24 h, the cells were harvested using trypsin without EDTA, then the cells were washed twice with ice-cold phosphate buffered saline (PBS) containing 2% FBS. The cells were then centrifuged, resuspended in 195 μL binding buffer on ice, add 5 μL FITC stained Annexin V, incubate 30 min for 15 min at 4 °C, and just before the analyzed, 5 μL PI was added. The data acquisition and analysis were performed using a FACS cytometer (FACS, CA, USA). A total of 1 × 10^5^ cells were scanned in each analysis. Each experiment was repeated at least three times.

### Statistical analysis

Data were compared using one-way ANOVA with posttest Newman-Keuls multiple comparison. *P* value of or less than 0.05 was considered statistically significant. The data are expressed as mean values ± SD/SEM and statistics were calculated with Graphpad Prism Software.

## Results

### Astaxanthin significantly ameliorated the cytotoxicity and apoptosis induced by cobalt treatment

**Fig. 1:**
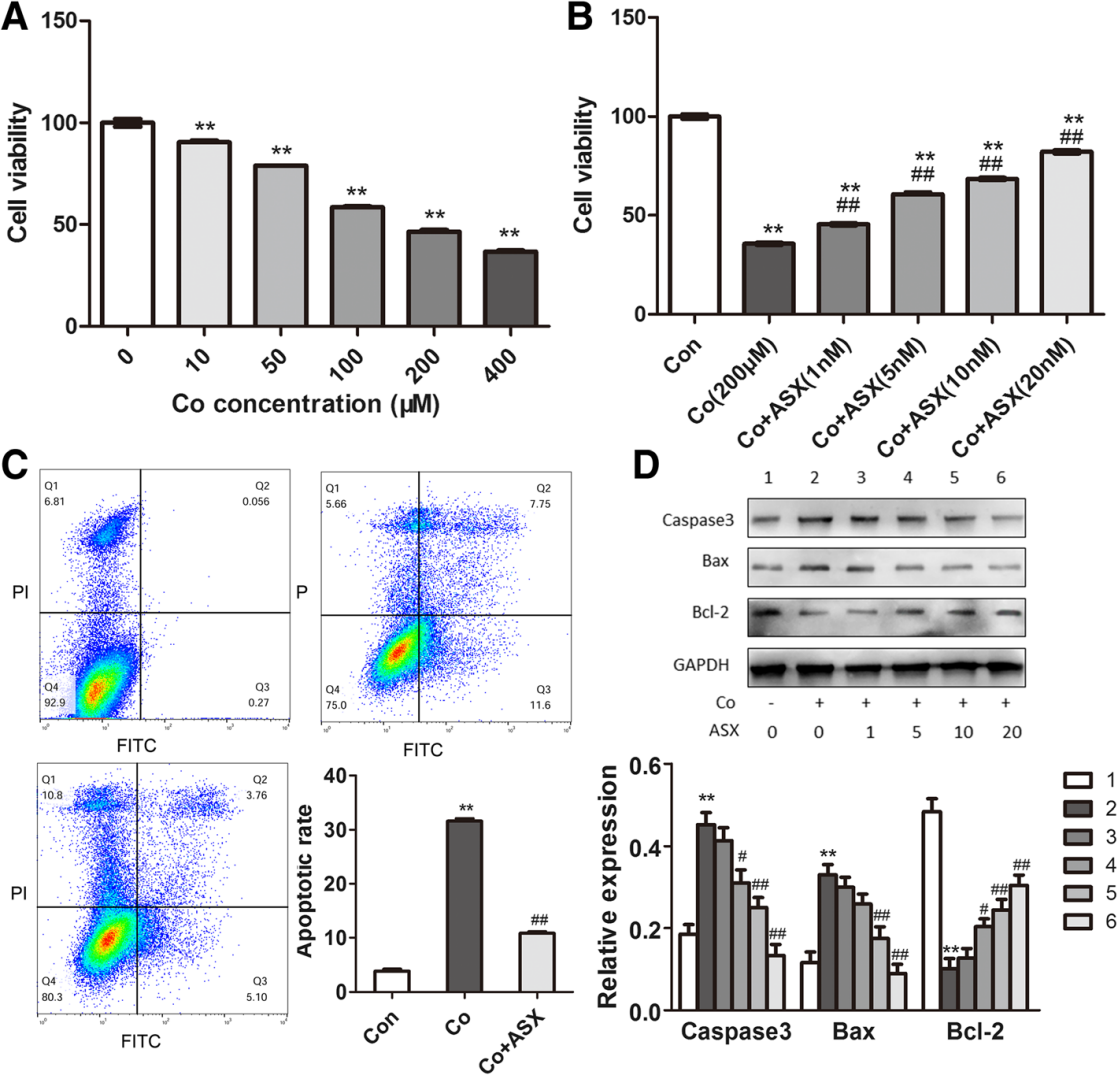
Astaxanthin significantly ameliorated cobalt-induced cytotoxicity and apoptosis. **a** The viability of MG-63 cells decreased as the concentration of Co^2+^ increases from 10 μM to 400 μM. (**P < 0.01 vs 0 μM) **b**) ASX significantly inhibited the cobalt cytotoxicity in the cell viability. [**P < 0.01 vs Con; ^##^ *P* < 0.01 vs Co (200)] **c**) FACS analysis showed that ASX (20 nM) significantly inhibited the cell apoptosis induced by Co^2+^exposure. **d** Western blot assay demonstrated that ASX (20 nM) significantly regulated related proteins related to apoptosis: compared to Co^2+^-exposed group, ASX-treated group exhibited significantly increased bcl-2 expression, decreased caspase-3 and bax expression. (1, vehicle-treated MG-63 cells; 2, Co^2+^-exposed cells; 3, Co^2+^-exposed cells treated by ASX (1 nM); 4, Co^2+^-exposed cells treated by ASX (5 nM); 5, Co^2+^-exposed cells treated by ASX (10 nM); 6, Co^2+^-exposed cells treated by ASX (20 nM); **P < 0.01 vs 1; ^##^ *P* < 0.01 vs 2)

First, we observed the cytotoxic effect of Co^2+^ (10, 50, 100, 200, 400 μM) treatment (24 h) in the viability of human MG-63 cells. As shown in Fig. 1, Co^2+^ treatment significantly reduced the cell viability as the concentration increased from 50 μM to 400 μM (Fig. 1a, Additional file 1: Table S1). Especially, at the concentration of 200 μM, Co^2+^ decreased the cell viability by about 50% (Fig. 1a); hence we chose 200 μM as the designated concentration in our subsequent experiment. Results showed that ASX treatment significantly enhanced the MG-63 cell viability (Additional file 2: Figure S1A). Compared to Co^2+^-treated group, the Co^2+^-exposed MG-63 cells treated by ASX exhibited a significant increase in the cell viability, as the concentration of ASX increased from 1 nM to 20 nM (Fig. 1b, Additional file 1: Table S2). To investigate the mechanism underlying the protective effect of ASX, we examined the apoptosis after Co^2+^ exposure and ASX treatment. It was found by FACS assay that ASX treatment significantly inhibited the MG-63 cell apoptosis induced by Co^2+^ exposure (Fig. 1c). Then we examined the expression of apoptosis-related target to explore the mechanism underlying the protective effect of ASX against the cell apoptosis. Results showed that, exposure to Co^2+^ for 24 h induced a significantly increased expression of Caspase-3 and Bax, a marked decreased expression of Bcl-2, in MG-63 cells; ASX pretreatment for 24 h remarkably attenuated Co^2+^-induced activation of apoptotic proteins (Fig. 1d).

### Astaxanthin attenuates the oxidative stress caused by cobalt exposure

**Fig. 2:**
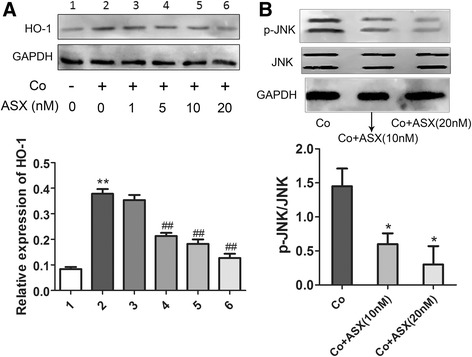
Protection of astaxanthin against cobalt cytotoxicity was related to HO-1 expression and JNK phosphorylation. **a** Compared to control, Co^2+^exposure significantly elevated the expression of HO-1, which was reduced by ASX pretreatment as the concentration rises. (1, vehicle-treated MG-63 cells; 2, Co^2+^-exposed cells; 3, Co^2+^-exposed cells treated by ASX (1 nM); 4, Co^2+^-exposed cells treated by ASX (5 nM); 5, Co^2+^-exposed cells treated by ASX (10 nM); 6, Co^2+^-exposed cells treated by ASX (20 nM); **P < 0.01 vs 1; ^##^ *P* < 0.01 vs 2) **b**) Compared to Co^2+^-exposed group, the ASX pretreatment significantly inhibited the phosphorylation of JNK. (**P < 0.05 vs Co)

Cobalt could promote cell apoptosis though stimulating oxidative stress and downstream mediators [31–33]. Therefore, we measured the intracellular ROS level using the fluorescent probe 2′,7′-dichlorofluorescin diacetate (DCFH/DA). It was shown that Co^2+^ significantly increased the DCF fluorescence intensity, while ASX markedly reduced the ROS level as the concentration rises from 1 nM to 20 nM (Additional file 2: Figure S1A). HO-1 is a generalized marker enzyme of antioxidant [30, 33]. It was found that ASX treatment for 24 h significantly decreased the expression of HO-1 (Fig. 2a). JNK pathway was reported by many to be related in the HO-1 induction in the oxidative stress [34–37]. We also observed a significant inhibition of JNK phosphorylation by ASX treatment. As oxidative stress plays an important role in cobalt-induced cytotoxicity and cell apoptosis, the protective effect of ASX might be attributed to its antioxidant capacity. Besides, we also observed an involvement of p38, ERK and AKT pathway in the protective effect of ASX against cobalt cytotoxicity (Additional file 2: Figure S1B).

### Downregulation of JNK did not affect the protective effect of astaxanthin against cobalt cytotoxicity

**Fig. 3:**
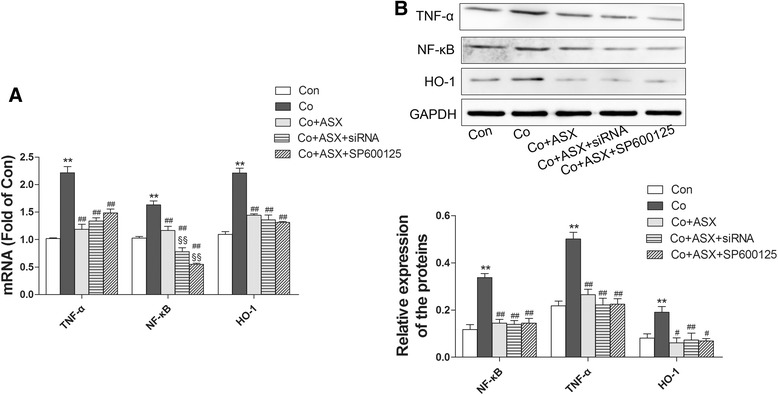
Downregulation of JNK did not significantly affect the protection of ASX against cobalt cytotoxicity. **a** qRT-PCR analysis showed that: ASX pretreatment markedly decreased the mRNA levels of TNF-α, NF-κB and HO-1, and downregulation of JNK by siRNA interfering or the inhibitor SP600125 did not significantly abolish the protection of ASX pretreatment. (**P < 0.01 vs Con; ^##^ *P* < 0.01 vs Co; ^§§^ *P* < 0.01 vs Co + ASX) **b**) Western blot assay showed that ASX pretreatment markedly decreased the expression of TNF-α, NF-κB and HO-1, and downregulation of JNK by siRNA interfering or the inhibitor SP600125 did not significantly abolish the protection of ASX pretreatment. (**P < 0.01 vs Con; ^##^ *P* < 0.01 vs Co; ^§§^ *P* < 0.01 vs Co + ASX)

Then we examined whether downregulation of JNK by siRNA or an inhibitor (SP600125) could affect the protective effect of ASX against cobalt cytotoxicity in MG-63 cells. At 24 h after siRNA interfering, qPCR analysis showed that the mRNA levels of TNF-α, NF-κB and HO-1 were significantly suppressed by ASX treatment (Fig. 3a). In addtion, downregulation of JNK did not significantly alter the mRNA levels of TNF-α and HO-1 compared to ASX-treated group; however, JNK suppression significantly enhanced the NF-κB mRNA level (Fig. 3a). Subsequent western blot analysis demonstrated that JNK downregulation did not alter the expression of TNF-α, NF-κB and HO-1 compared to ASX-treated group (Fig. 3b).

## Discussion

The current study found that a dietary supplement ASX might have a protective effect against cobalt toxicity in MG-63 cells, implying a possible application of ASX in patients who underwent metal-metal hip articulation.

Cobalt is a mineral which is required as dietary supplements in trace amount. But it displays toxic effects when present at high concentration. Groups of patients implanted with hip devices of MoM bearing surface have been shown to develop an accelerated inflammatory reaction frequently associated with tissue necrosis and cellular toxicity; because of tribocorrosion, metal-based implants release wear debris. And the particles released from the implants contain Co^2+^ [38]. It has been reported that Co^2+^ could induce monocyte/macrophages to release bone absorption factor and cause bone around the prosthesis dissolved [39], 100 μM Co^2+^ resulted in significant decreases in cell viability accompanied by a significant increase in apoptosis on primary human lymphocytes, Co^2+^ also have a cytotoxic effect on human MG-63 osteoblasts cell [38], our results confirmed that Co^2+^ is toxic to MG-63 cells.

ASX is a carotenoid that possesses strong antioxidant activity [40]. It was reported that the parameters of ASX were dose dependent if administrated intravenously, but dose independent if taken orally, due to primary metabolizm by hepatic cytochrome P450 [41]. Besides, diet could elevate the bioavailability of ASX significantly [42]. Recent studies reported that ASX could alleviate oxidative stress in vitro and vivo. In human vascular endothelial cells, it would attenuate glucose fluctuation-caused oxidative stress and cell apoptosis [43]; in vivo, ASX was showed to significantly ameliorate hepatic ischemia reperfusion (IR) injury by reducing ROS level and inhibiting MAPK pathway [44]; the underlying mechanism was partly involved with the downregulation of NF-κB activity [45, 46]. The present study demonstrated that ASX treatment markedly inhibited the cobalt toxicity in MG-63 cells, which could be partially attributed to oxidative stress pathway.

Our research explored the antidote effect of ASX against Co toxicity in the way of oxidative stress and related pathways, such as JNK, HO-1, TNF-α, NF-κB etc. Extensive reports showed that ROS was closely related to JNK, P38 and ERKs pathway [47, 48]. These findings are consistent with our results. Mitani et al. [49] demonstrated that induction of HO-1 expression could inhibit inflammatory reaction through decreasing oxidative stress in mice intestine. Preventing JNK, P38 activation and mitochondrial pathway could ameliorate high glucose-induced PC12 cell apoptosis [50]. All the evidences highlighted the role of oxidative stress in the mechanism by which ASX ameliorated cobalt toxicity in MG-63 cells.

## Conclusion

In conclusion, our results show that increase in cobalt (II) concentration could induce significant apoptosis in MG-63 cells, which could be inhibited by ASX treatment. The underlying mechanism was probably involved with modulation in oxidative stress. Whether ASX can be applied in clinical to reduce the MoM artificial hip caused side-effect needs further in-vivo study.

## Additional files

Additional file 1: Table S1. The results of Figure 1a. **Table S2.** The results of Figure 2b. (DOCX 12 kb)

Additional file 2: Figure S1. A) The MG-63 cell viability was significantly improved by ASX treatment. (**P < 0.01 vs 0); B) The ROS level of the Co^2+^-exposed cells treated by ASX significantly decreased compared to Co^2+^-exposed group, as the ASX concentration increased. (**P < 0.01 vs Con; ^##^ *P* < 0.01 vs Co) C) The P38, ERK, and AKT pathway were involved in the protective effect of ASX against cobalt cytotoxicity. (TIFF 1382 kb)

## Abbreviations

ASX: Astaxanthin
ATCC: American Type Culture Collection
CSF: Colony stimulating factor
HO-1: Heme oxygenase 1
FACS: Flourescence-activated cell sorting
JNK: c-Jun N-terminal Kinase
MAPK: Mitogen-activated protein kinase
MoM: Metal-on-metal
RANKL: Receptor Activator for Nuclear Factor-κB Ligand
ROS: Reactive oxygen species
THP: Total hip replacement

## References

[1] Learmonth ID, Young C, Rorabeck C (2007). The operation of the century: total hip replacement. Lancet.

[2] Innmann MM, Gotterbarm T, Kretzer JP, Merle C, Ewerbeck V, Weiss S, Aldinger PR, Streit MR (2014). Minimum ten-year results of a 28-mm metal-on-metal bearing in cementless total hip arthroplasty in patients fifty years of age and younger. Int Orthop.

[3] Smith SL, Dowson D, Goldsmith AA (2001). The effect of femoral head diameter upon lubrication and wear of metal-on-metal total hip replacements. Proc Inst Mech Eng H.

[4] Cuckler JM, Moore KD, Lombardi AV, McPherson E, Emerson R (2004). Large versus small femoral heads in metal-on-metal total hip arthroplasty. J Arthroplast.

[5] Dorr LD, Long WT (2005). Metal-on-metal: articulations for the new millennium. Instr Course Lect.

[6] MacDonald SJ, McCalden RW, Chess DG, Bourne RB, Rorabeck CH, Cleland D, Leung F. Metal-on-metal versus polyethylene in hip arthroplasty: a randomized clinical trial. Clin Orthop Relat Res. 2003;406:282–96. 10.1097/01.blo.0000043066.62337.9d 12579029

[7] Ishida T, Clarke IC, Donaldson TK, Shirasu H, Shishido T, Yamamoto K (2009). Comparing ceramic-metal to metal-metal total hip replacements--a simulator study of metal wear and ion release in 32- and 38-mm bearings. J Biomed Mater Res B Appl Biomater.

[8] Milosev I, Pisot V, Campbell P (2005). Serum levels of cobalt and chromium in patients with Sikomet metal-metal total hip replacements. J Orthop Res.

[9] Hartmann A, Hannemann F, Lutzner J, Seidler A, Drexler H, Gunther KP, Schmitt J (2013). Metal ion concentrations in body fluids after implantation of hip replacements with metal-on-metal bearing--systematic review of clinical and epidemiological studies. PLoS One.

[10] Bradberry SM, Wilkinson JM, Ferner RE (2014). Systemic toxicity related to metal hip prostheses. Clin Toxicol (Phila).

[11] Liu YK, Ye J, Han QL, Tao R, Liu F, Wang W (2015). Toxicity and bioactivity of cobalt nanoparticles on the monocytes. Orthop Surg.

[12] Vendittoli PA, Amzica T, Roy AG, Lusignan D, Girard J, Lavigne M (2011). Metal ion release with large-diameter metal-on-metal hip arthroplasty. J Arthroplast.

[13] Witzleb WC, Ziegler J, Krummenauer F, Neumeister V, Guenther KP (2006). Exposure to chromium, cobalt and molybdenum from metal-on-metal total hip replacement and hip resurfacing arthroplasty. Acta Orthop.

[14] Simonsen LO, Harbak H, Bennekou P (2012). Cobalt metabolism and toxicology--a brief update. Sci Total Environ.

[15] Scharf B, Clement CC, Zolla V, Perino G, Yan B, Elci SG, Purdue E, Goldring S, Macaluso F, Cobelli N (2014). Molecular analysis of chromium and cobalt-related toxicity. Sci Rep.

[16] Akbar M, Brewer JM, Grant MH (2011). Effect of chromium and cobalt ions on primary human lymphocytes in vitro. J Immunotoxicol.

[17] Kwon YM, Xia Z, Glyn-Jones S, Beard D, Gill HS, Murray DW (2009). Dose-dependent cytotoxicity of clinically relevant cobalt nanoparticles and ions on macrophages in vitro. Biomed Mater.

[18] Lohmann CH, Schwartz Z, Koster G, Jahn U, Buchhorn GH, MacDougall MJ, Casasola D, Liu Y, Sylvia VL, Dean DD, Boyan BD (2000). Phagocytosis of wear debris by osteoblasts affects differentiation and local factor production in a manner dependent on particle composition. Biomaterials.

[19] Jost-Albrecht K, Hofstetter W (2006). Gene expression by human monocytes from peripheral blood in response to exposure to metals. J Biomed Mater Res B Appl Biomater.

[20] Mandelin J, Li TF, Liljestrom M, Kroon ME, Hanemaaijer R, Santavirta S, Konttinen YT (2003). Imbalance of RANKL/RANK/OPG system in interface tissue in loosening of total hip replacement. J Bone Joint Surg Br.

[21] Higuera-Ciapara I, Felix-Valenzuela L, Goycoolea FM (2006). Astaxanthin: a review of its chemistry and applications. Crit Rev Food Sci Nutr.

[22] Pashkow FJ, Watumull DG, Campbell CL (2008). Astaxanthin: a novel potential treatment for oxidative stress and inflammation in cardiovascular disease. Am J Cardiol.

[23] Naguib YM (2000). Antioxidant activities of astaxanthin and related carotenoids. J Agric Food Chem.

[24] Song X, Wang B, Lin S, Jing L, Mao C, Xu P, Lv C, Liu W, Zuo J (2014). Astaxanthin inhibits apoptosis in alveolar epithelial cells type II in vivo and in vitro through the ROS-dependent mitochondrial signalling pathway. J Cell Mol Med.

[25] Ambati RR, Phang SM, Ravi S, Aswathanarayana RG (2014). Astaxanthin: sources, extraction, stability, biological activities and its commercial applications--a review. Mar Drugs.

[26] Barim-Oz O, Sahin H (2016). The influence of dietary antioxidant on ovarian eggs and levels of vitamin E, C, a, astaxanthin, beta-carotene and oxidative stres in tissues of Astacus Leptodactylus (Eschscholtz) during reproduction. Cell Mol Biol (Noisy-le-grand).

[27] Fang Q, Guo S, Zhou H, Han R, Wu P, Han C (2017). Astaxanthin protects against early burn-wound progression in rats by attenuating oxidative stress-induced inflammation and mitochondria-related apoptosis. Sci Rep.

[28] Kim B, Farruggia C, Ku CS, Pham TX, Yang Y, Bae M, Wegner CJ, Farrell NJ, Harness E, Park YK (2016). Astaxanthin inhibits inflammation and fibrosis in the liver and adipose tissue of mouse models of diet-induced obesity and nonalcoholic steatohepatitis. J Nutr Biochem.

[29] Lin KH, Lin KC, Lu WJ, Thomas PA, Jayakumar T, Sheu JR. Astaxanthin, a carotenoid, stimulates immune responses by enhancing IFN-gamma and IL-2 secretion in primary cultured lymphocytes in vand ex vivo. Int J Mol Sci. 2015;17:44. 10.3390/ijms17010044 PMC4730289 26729100

[30] Fleury C, Petit A, Mwale F, Antoniou J, Zukor DJ, Tabrizian M, Huk OL (2006). Effect of cobalt and chromium ions on human MG-63 osteoblasts in vitro: morphology, cytotoxicity, and oxidative stress. Biomaterials.

[31] Hsu H, Shu HB, Pan MG, Goeddel DV (1996). TRADD-TRAF2 and TRADD-FADD interactions define two distinct TNF receptor 1 signal transduction pathways. Cell.

[32] Rothe M, Wong SC, Henzel WJ, Goeddel DV (1994). A novel family of putative signal transducers associated with the cytoplasmic domain of the 75 kDa tumor necrosis factor receptor. Cell.

[33] Micheau O, Tschopp J (2003). Induction of TNF receptor I-mediated apoptosis via two sequential signaling complexes. Cell.

[34] Kietzmann T, Samoylenko A, Immenschuh S (2003). Transcriptional regulation of heme oxygenase-1 gene expression by MAP kinases of the JNK and p38 pathways in primary cultures of rat hepatocytes. J Biol Chem.

[35] Liang J, Xu H, Wu Y, Sun S, Jia Z, Wei C, You J (2009). Effect of serum from Overfatigue rats on JNK/c-Jun/HO-1 pathway in human umbilical vein endothelial cells and the intervening effect of Tongxinluo(通心络)superfine powder. Chin J Integr Med.

[36] Choe Y, Lee S, Ko KW, Shin SJ, Kim H (2014). Nutlin-3 induces HO-1 expression by activating JNK in a transcription-independent manner of p53. Int J Oncol.

[37] Lin C, Hsiao W, Huang CY, Kao C, Hsu GW (2013). Heme oxygenase-1 induction by the ROS-JNK pathway plays a role in aluminum-induced anemia. J Inorg Biochem.

[38] Yashima Y, Okamoto K, Sakai E, Iwatake M, Fukuma Y, Nishishita K, Tsukuba T (2015). Cobalt protoporphyrin represses osteoclastogenesis through blocking multiple signaling pathways. Biometals.

[39] Takayanagi H (2005). Mechanistic insight into osteoclast differentiation in osteoimmunology. J Mol Med (Berl).

[40] Wu H, Niu H, Shao A, Wu C, Dixon BJ, Zhang J, Yang S, Wang Y (2015). Astaxanthin as a potential Neuroprotective agent for neurological diseases. Mar Drugs.

[41] Choi HD, Kang HE, Yang SH, Lee MG, Shin WG (2011). Pharmacokinetics and first-pass metabolism of astaxanthin in rats. Br J Nutr.

[42] Okada Y, Ishikura M, Maoka T (2009). Bioavailability of astaxanthin in Haematococcus algal extract: the effects of timing of diet and smoking habits. Biosci Biotechnol Biochem.

[43] Abdelzaher LA, Imaizumi T, Suzuki T, Tomita K, Takashina M, Hattori Y (2016). Astaxanthin alleviates oxidative stress insults-related derangements in human vascular endothelial cells exposed to glucose fluctuations. Life Sci.

[44] Li J, Wang F, Xia Y, Dai W, Chen K, Li S, Liu T, Zheng Y, Wang J, Lu W (2015). Astaxanthin pretreatment attenuates hepatic ischemia reperfusion-induced apoptosis and Autophagy via the ROS/MAPK pathway in mice. Mar Drugs.

[45] Yeh PT, Huang HW, Yang CM, Yang WS, Yang CH (2016). Astaxanthin inhibits expression of retinal oxidative stress and inflammatory mediators in Streptozotocin-induced diabetic rats. PLoS One.

[46] Al-Amin MM, Reza HM, Saadi HM, Mahmud W, Ibrahim AA, Alam MM, Kabir N, Saifullah AR, Tropa ST, Quddus AH (2016). Astaxanthin ameliorates aluminum chloride-induced spatial memory impairment and neuronal oxidative stress in mice. Eur J Pharmacol.

[47] Jia D, Lu W, Zhang X, Cai G, Teng L, Wang X, Zhang M, Zeng Y, Liang C, Wang D (2016). Calf spleen extractive injection (CSEI), a small peptides enriched extraction, induces human hepatocellular carcinoma cell apoptosis via ROS/MAPKs dependent mitochondrial pathway. J Pharmacol Sci.

[48] Yin H, Sun G, Yang Q, Chen C, Qi Q, Wang H, Li J (2017). NLRX1 accelerates cisplatin-induced ototoxity in HEI-OC1 cells via promoting generation of ROS and activation of JNK signaling pathway. Sci Rep.

[49] Mitani T, Yoshioka Y, Furuyashiki T, Yamashita Y, Shirai Y, Ashida H (2017). Enzymatically synthesized glycogen inhibits colitis through decreasing oxidative stress. Free Radic Biol Med.

[50] Aminzadeh A. Protective effect of tropisetron on high glucose induced apoptosis and oxidative stress in PC12 cells: roles of JNK, P38 MAPKs, and mitochondria pathway. Metab Brain Dis. 2017;32:819-26. 10.1007/s11011-017-9976-5 28243846

