# Supplementary material for: Astaxanthin mitigates cobalt cytotoxicity in the MG-63 cells by modulating the oxidative stress
Source: BMC Pharmacol Toxicol. 2017 Jul 24;18:58. doi: 10.1186/s40360-017-0166-1 (PMC5525213; doi:10.1186/s40360-017-0166-1)
Supplement: Supplementary file 1 — The results of Figure 1a. Table S2. The results of Figure 2b. (DOCX 12 kb) [file 40360_2017_166_MOESM1_ESM.docx]

|  | Mean | SD | n | *p* vs control |
| --- | --- | --- | --- | --- |
| Control(Co 0μM) | 100 | 1.913 | 3 | -- |
| Co 10μM | 90.48 | 1.543 | 3 | 0.0026 |
| Co 50μM | 78.92 | 0.2194 | 3 | <0.0001 |
| Co 100μM | 58.47 | 0.7404 | 3 | <0.0001 |
| Co 200μM | 40.42 | 1.579 | 3 | <0.0001 |
| Co 400μM | 32.54 | 1.082 | 3 | <0.0001 |

Supplementary table 1. The results of figure 1A.

|  | Mean | SD | n | *p* vs control | *p* vs Co 200μM |
| --- | --- | --- | --- | --- | --- |
| Control | 100 | 1.145 | 3 | -- | -- |
| Co 200μM | 37.56 | 0.6002 | 3 | <0.0001 | -- |
| Co 200μM +ASX 1nM | 47.44 | 0.6067 | 3 | <0.0001 | <0.0001 |
| Co 200μM +ASX 5nM | 61.70 | 1.040 | 3 | <0.0001 | <0.0001 |
| Co 200μM +ASX 10nM | 68.43 | 0.6067 | 3 | <0.0001 | <0.0001 |
| Co 200μM +ASX 20nM | 82.13 | 0.8179 | 3 | <0.0001 | <0.0001 |

Supplementary table 2. The results of figure 2B.
