# Supplementary figures and images for: Astaxanthin mitigates cobalt cytotoxicity in the MG-63 cells by modulating the oxidative stress
Source: BMC Pharmacol Toxicol. 2017 Jul 24;18:58. doi: 10.1186/s40360-017-0166-1 (PMC5525213; doi:10.1186/s40360-017-0166-1)

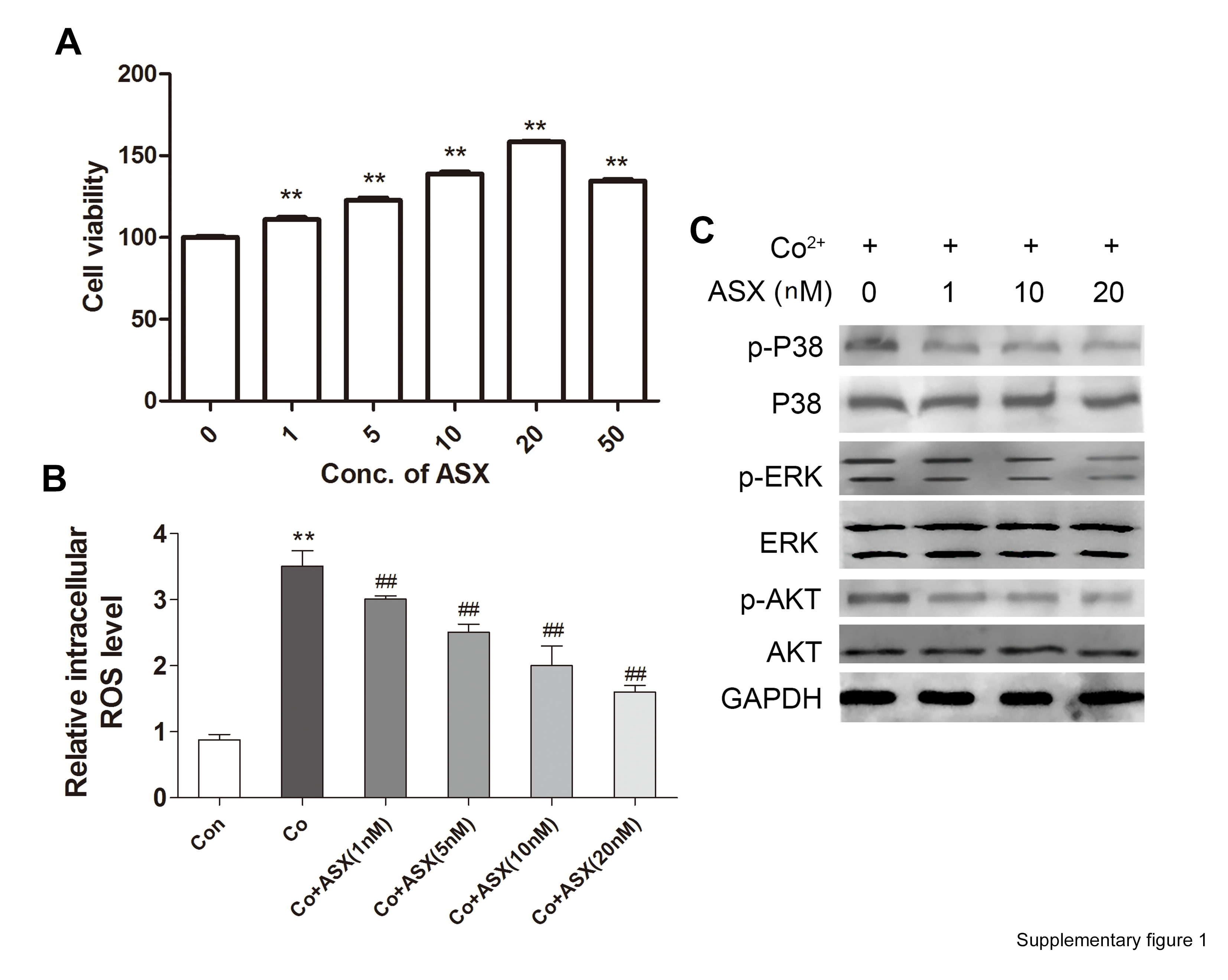

Supplement: Supplementary file 2 — A) The MG-63 cell viability was significantly improved by ASX treatment. (**P < 0.01 vs 0); B) The ROS level of the Co2+-exposed cells treated by ASX significantly decreased compared to Co2+-exposed group, as the ASX concentration increased. (**P < 0.01 vs Con; ## P < 0.01 vs Co) C) The P38, ERK, and AKT pathway were involved in the protective effect of ASX against cobalt cytotoxicity. (TIFF 1382 kb) [file 40360_2017_166_MOESM2_ESM.tif]
